# Preventing First and Further Decompensation in Advanced Chronic Liver Disease

**DOI:** 10.1111/liv.70568

**Published:** 2026-03-03

**Authors:** Leonardo Corrêa Süffert, Bernardo de Faria Moraes, Guilherme Grossi Lopes Cançado

**Affiliations:** ^1^ School of Medicine Pontifícia Universidade Católica Do Rio Grande Do Sul Porto Alegre Brazil; ^2^ Universidade Federal de Minas Gerais Belo Horizonte Brazil; ^3^ Hospital das Clínicas da Universidade Federal de Minas Gerais Belo Horizonte Brazil; ^4^ Department of Gastroenterology and Hepatology Hospital da Polícia Militar de Minas Gerais Belo Horizonte Brazil

**Keywords:** ascites, haemorrhage, hepatic encephalopathy, liver cirrhosis, recompensation, TIPS

## Abstract

Advanced chronic liver disease (ACLD) remains a major cause of global morbidity and mortality. Preventing hepatic decompensation—both the first event and subsequent recurrences—has become a central therapeutic goal to prolong survival. The transition from the compensated phase (cACLD) to the decompensated phase (dACLD) is driven by clinically significant portal hypertension (CSPH) and continuous exposure to etiologic factors, and is often precipitated by systemic triggers such as infections, portal vein thrombosis, and hepatocellular carcinoma. Thus, effective prevention requires a multidisciplinary strategy combining etiologic control with hemodynamic modulation, supported by vaccination, optimized nutrition and physical activity, judicious endoscopic therapy, and a critical reassessment of antibiotic prophylaxis in the era of antimicrobial resistance. Among non‐selective beta‐blockers (NSBBs), carvedilol—through combined β1/β2 and α1 blockade—achieves a greater reduction in hepatic venous pressure gradient (HVPG) than propranolol and demonstrates superiority in preventing first decompensation. In dACLD, although the effect of NSBBs is attenuated, carvedilol still emerges as the preferred option, given that propranolol shows significantly lower efficacy at this stage. Endoscopic variceal ligation (EVL) remains an alternative for NSBB‐intolerant patients in cACLD and is essential for secondary prophylaxis. Moreover, in dACLD, the combination of EVL with carvedilol is increasingly being explored in Child‐Pugh B/C patients. We provide an overview of pathophysiological mechanisms, risk stratification using non‐invasive tests, and pragmatic prevention strategies across the different stages of disease, emphasizing recompensation, the NSBB “therapeutic window,” and the need to revisit routine antibiotic prophylaxis.

## Key Points

- Preventing first and subsequent decompensation in advanced chronic liver disease is challenging.
- Treating the etiologic cause is the main objective and should be followed by multidisciplinary strategies at the time of diagnosis.

**Abbreviations**
ACLD: Advanced chronic liver disease
ACLF: Acute‐on‐chronic liver failure
AUC: Area Under the Curve
cACLD: Compensated advanced chronic liver disease
CSPH: Clinically significant portal hypertension
dACLD: Decompensated advanced chronic liver disease
EVL: Endoscopic variceal ligation
HCC: Hepatocellular carcinoma
HE: Hepatic encephalopathy
HRS: Hepatorenal syndrome
HRS‐AKI: Hepatorenal syndrome–acute kidney injury
HVPG: Hepatic venous pressure gradient
IVR: Intrahepatic vascular resistance
LSM: Liver stiffness measurement
MASH: Metabolic dysfunction–associated steatohepatitis
MASLD: Metabolic dysfunction–associated steatotic liver disease
NSBBs: Non‐selective beta‐blockers
PH: Portal hypertension
RCT: Randomised controlled trial
SBP: Spontaneous bacterial peritonitis
TIPS: Transjugular intrahepatic portosystemic shunt
VB: Variceal bleeding

## 1 Introduction

Cirrhosis represents the final stage of various chronic liver diseases and remains a major cause of morbidity and mortality worldwide. Its global prevalence is estimated at approximately 1.3% [1]. In 2019, about 1.47 million deaths were directly attributed to cirrhosis and other chronic liver diseases, representing a 45% increase in absolute numbers since 1990 [2]. As hepatitis B and C cases have declined with preventive and antiviral advances, metabolic dysfunction–associated steatohepatitis (MASH) and alcohol abuse have emerged as the leading causes of cirrhosis, particularly in developed countries [1, 3].

Histopathological diagnosis of cirrhosis is not always feasible in clinical practice [4, 5, 6]. Therefore, recent guidelines, such as Baveno VII [6] and the American Association for the Study of Liver Diseases (AASLD) 2024 [5], recommend the use of the term: advanced chronic liver disease (ACLD) to encompass the full spectrum of advanced liver disease, including patients who are highly likely to have cirrhosis even in the absence of histological confirmation [5, 6]. This change in terminology aims to overcome the limitations of a purely histopathological definition by incorporating clinical and non‐invasive criteria, such as hepatic elastography techniques, which assess liver stiffness (liver stiffness measurement—LSM) and correlate with the degree of fibrosis and portal hypertension (PH). In this context, LSM < 10 kPa reliably excludes ACLD; values between 10 to 15 kPa are suggestive, 15–20 kPa are highly suggestive, and values > 20 kPa confirm ACLD [4, 5, 6].

PH is the main determinant of morbidity and mortality in ACLD, driving hepatic decompensations such as variceal bleeding (VB), hepatic encephalopathy (HE), and ascites. ACLD is broadly classified into compensated (cACLD) and decompensated (dACLD) stages, with the transition marked by the onset of these events—a direct consequence of clinically significant portal hypertension (CSPH), defined by a hepatic venous pressure gradient (HVPG) ≥ 10 mmHg [4, 5, 6]. Preventing decompensation is therefore essential to improve survival. However, therapeutic decisions must consider several nuances that vary across ACLD stages, including the choice of non‐selective beta‐blockers (NSBBs), the role of endoscopic variceal ligation (EVL), etiological treatment, nutritional management, vaccination, and antibiotic prophylaxis. This review aims to critically and comprehensively evaluate the main strategies to prevent hepatic decompensation in ACLD, integrating the most relevant evidence and offering updated insights on preventing both the first and further decompensation.

## 2 Mechanisms and Predictors of Hepatic Decompensation in Portal Hypertension

PH arises from increased intrahepatic vascular resistance (IVR) plus augmented portal inflow [7]. The structural component of IVR reflects fibrosis, nodular regeneration, sinusoidal capillarization, and microthrombosis [8, 9]; the dynamic component (~30% of IVR) derives from perisinusoidal cell contraction and endothelial dysfunction with reduced nitric‐oxide bioavailability and excess vasoconstrictors [7, 8, 10]. Importantly, the relative contribution and biological drivers of the dynamic component are aetiology‐dependent, varying according to the predominant pattern of liver injury, inflammation, cholestasis, and vascular remodelling—thereby underscoring that the risk of hepatic decompensation is not uniform, but rather varies across different cirrhosis etiologies [11, 12]. As portal pressure rises, splanchnic vasodilatation, angiogenesis, and collateral formation create a hyperdynamic state that sustains CSPH and drives clinical events [10, 13, 14].

**FIGURE 1:**
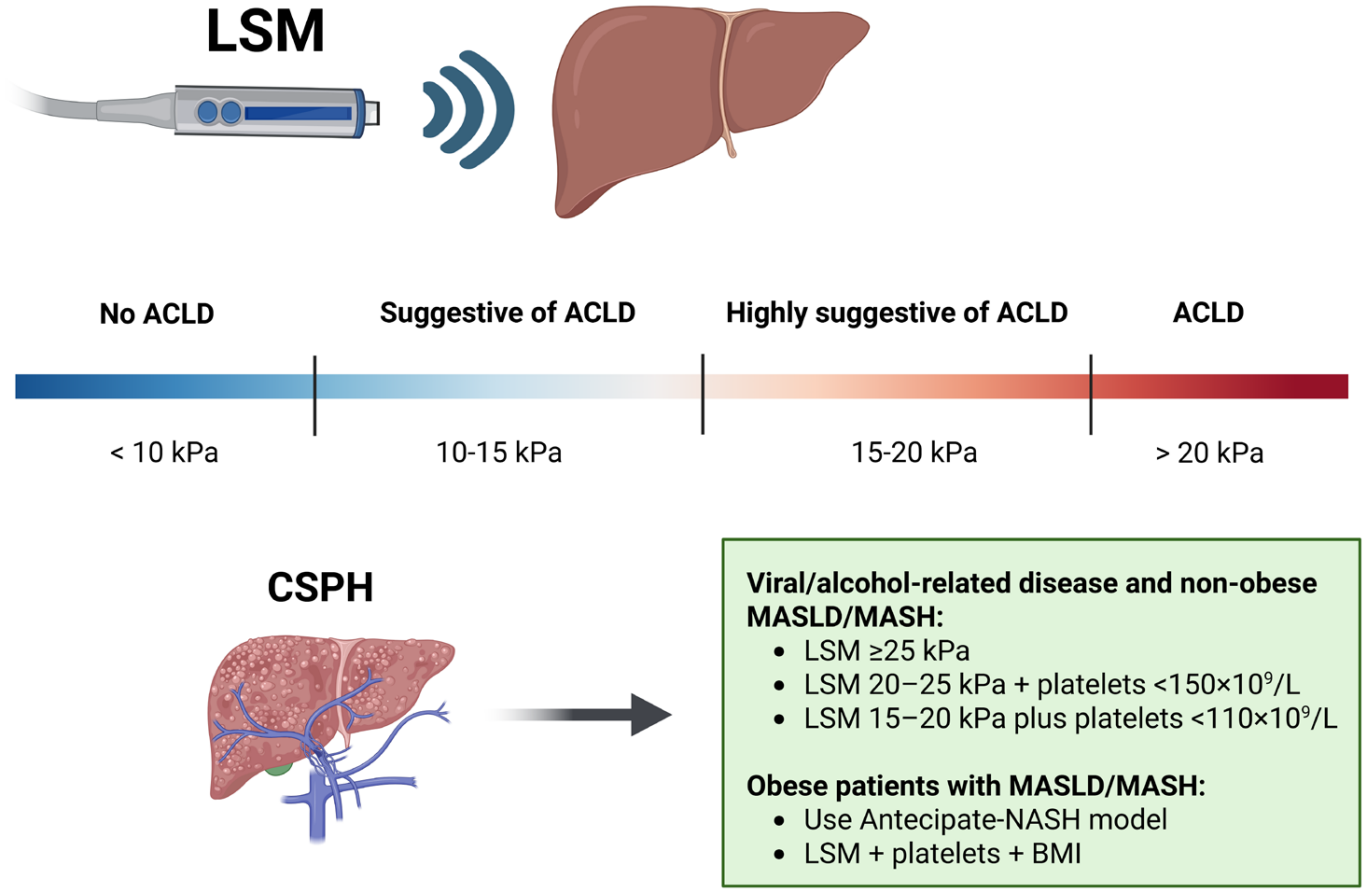
Threshold‐based interpretation of LSM for ACLD and CSPH. ACLD (Advanced Chronic Liver Disease), BMI (Body Mass Index), CSPH (Clinically Significant Portal Hypertension), LSM (Liver Stiffness Measurement), MASLD (Metabolic Dysfunction–Associated Steatotic Liver Disease), and MASH (Metabolic Dysfunction–Associated Steatohepatitis), and NASH (Nonalcoholic Steatohepatitis).

Non‐invasive tests now approximate HVPG for prognostication [15]. CSPH can be ruled out when LSM ≤ 15 kPa and platelets ≥ 150 × 10^9^/L [5, 6]. Pragmatically, it can be inferred in viral/alcohol‐related disease and non‐obese metabolic dysfunction–associated steatotic liver disease (MASLD) when LSM ≥ 25 kPa; with LSM 20–25 kPa plus platelets < 150 × 10^9^/L; LSM 15–20 kPa plus platelets < 110 × 10^9^/L [5, 6]. In obesity with MASH, the positive predictive value of LSM ≥ 25 kPa is lower [16]; the ANTICIPATE‐NASH model (LSM, platelets, Body Mass Index) improves accuracy and outperforms histology for liver event prediction [5, 16, 17] (Figure 1). When elastography is unavailable, endoscopic surveillance is recommended unless imaging already reveals portosystemic collaterals [5, 6]. Splenic stiffness (> 40 kPa) further refines CSPH diagnosis when LSM is indeterminate [18, 19]. Most recently, the addition of spleen stiffness measurement to LSM, body mass index, and platelet count (NICER model) yielded a significantly higher Area Under the Curve (AUC) for predicting CSPH than the ANTICIPATE‐NASH model (AUC 0.889 [0.843–0.934] vs. 0.849 [0.794–0.903]; *p* = 0.022) in risk stratification of patients with Child–Pugh class A cACLD [19]. Similarly, the VITRO score also demonstrated good predictive performance for the detection of CSPH (AUC 0.909 [0.823–0.965]) among patients who were ‘unclassified’ by the Baveno VII criteria [20].

Once dACLD develops, vasodilation and arterial underfilling activate neurohumoral systems, promoting sodium retention, ascites, and impaired renal perfusion, with risk of hepatorenal syndrome–acute kidney injury (HRS‐AKI) [21, 22]. Gut dysbiosis and immune dysfunction predispose to spontaneous bacterial peritonitis (SBP) [23, 24]. Gastroesophageal varices emerge as adaptive shunts to PH, expanding through dilation and angiogenesis; as pressure rises, their walls thin and may rupture, causing haemorrhage [25, 26]. Moreover, cirrhosis induces HE from ammonia accumulation, oxidative stress, and neuroinflammation disrupting neurotransmission and cerebral homeostasis [4, 27, 28]. Clinically, patients with dACLD can be subdivided according to the nature of decompensation into two major groups: those with acute decompensation, defined by rapid deterioration of the clinical condition, often requiring non‐elective/emergency hospitalisation due to the severity of the event, frequently progressing toward Acute‐on‐Chronic Liver Failure (ACLF), and those with non‐acute decompensation, typically characterised by a slow and progressive development of complications, manageable in an outpatient setting [4, 29].

## 3 Prevention of the First Decompensation

The compensated phase often spans a decade with annual mortality of approximately 1.5% [4]. After CSPH develops, the untreated risk of first decompensation approximates 5% per year [4]. The first decompensating event can present as (1) bleeding only; (2) non‐bleeding (ascites or HE); or (3) combined bleeding and non‐bleeding, with 1‐year mortality roughly 10%–16%, 9%–19%, and ~30%, respectively [4]. Patients at first decompensation may show a single decompensation event in 58%–72% of cases, most commonly the formation of ascites. Ascites must reach grade 2 or higher (overt ascites) to be considered a decompensating event. Similarly, HE must present as at least grade 2 or higher of West Haven [4, 5]. Regarding jaundice, its classification as a decompensating event remains debated. According to Baveno VII, non‐cholestatic jaundice marks the onset of dACLD and is associated with worse prognosis. However, other authors argue that current evidence is insufficient to regard jaundice alone as a true decompensation event [4, 6].

### 3.1 Triggers of Decompensation, Etiological Treatment and Vaccination

Preventable triggers include ongoing alcohol use, uncontrolled metabolic risk (obesity, diabetes), malnutrition and sarcopenia, major surgery without optimization, infection, viral hepatitis, HCC, portal vein thrombosis, and uncontrolled CSPH [4]. However, the most important goal is to treat the underlying aetiology of liver disease—such as cure of HCV, suppression of HBV, or sustained alcohol abstinence—as this reduces disease progression and lowers the risk of decompensation [4, 5, 6]. Vaccination against Influenza, Pneumococcus, SARS‐CoV‐2, and hepatitis A/B are essential, as these vaccines have proven to be very effective in reducing severe outcomes and mortality in this high‐risk population [30]. Nutritional and exercise interventions mitigate sarcopenia and systemic inflammation: target protein 1.2–1.5 g/kg/day, avoid prolonged fasting, and use a late‐evening snack [31, 32]. Biannual ultrasound surveillance facilitates early detection of HCC and portal vein thrombosis, enabling timely therapy [5].

### 3.2 NSBBs in the Management of PH in cACLD

Lebrec et al. [33] conducted the first randomised controlled trial (RCT) to demonstrate the efficacy of NSBBs in preventing VB, a finding that was subsequently confirmed by multiple studies. More recently, the PREDESCI trial [34] showed that NSBBs also reduce the risk of first decompensation in patients with cACLD and CSPH, mainly by lowering the incidence of ascites (HR: 0.44; 95% CI: 0.20–0.97; *p* = 0.0297) through a reduction in HVPG. Among NSBBs, carvedilol is the most effective [5, 6, 35], as it blocks β₁‐, β₂‐, and α₁‐receptors, thereby reducing cardiac output and splanchnic flow while promoting intrahepatic vasodilation [34]. This combined mechanism enhances HVPG reduction, lowering the risk of first decompensation and mortality [5, 6, 35, 36, 37], with optimal dosing at 12.5 mg daily [6, 35]. NSBBs are generally safe in patients with cACLD; however, adverse effects such as hypotension and bradycardia may occur, requiring treatment discontinuation [6, 38]. Notably, not all patients respond to NSBBs, and reliable non‐invasive predictors of hemodynamic response remain unavailable. Patient selection for NSBB therapy relies on identifying CSPH, as benefit is limited to this population. Controversy remains regarding NSBB use in patients without CSPH, as robust data supporting benefits in this group are lacking [39]. In this scenario, management should focus on etiological treatment to halt liver injury, reduce intrahepatic resistance, and prevent further increases in PH [5, 6, 39].

### 3.3 The Role of EVL in cACLD

Despite its good efficacy and safety profile in the primary prophylaxis of VB, EVL in patients with cACLD is generally reserved for those who are intolerant to NSBBs. This is because NSBBs exert effects beyond the prevention of VB, also reducing the risk of first decompensation and ascites [5, 6, 34]. Although EVL may outperform some NSBBs like propranolol [40], its effect is limited to variceal eradication. Nevertheless, in contraindications to NSBBs, endoscopy screening should be performed if LSM is ≥ 20 kPa or platelet count is ≤ 150 × 10^9^/L^6^.

**FIGURE 2:**
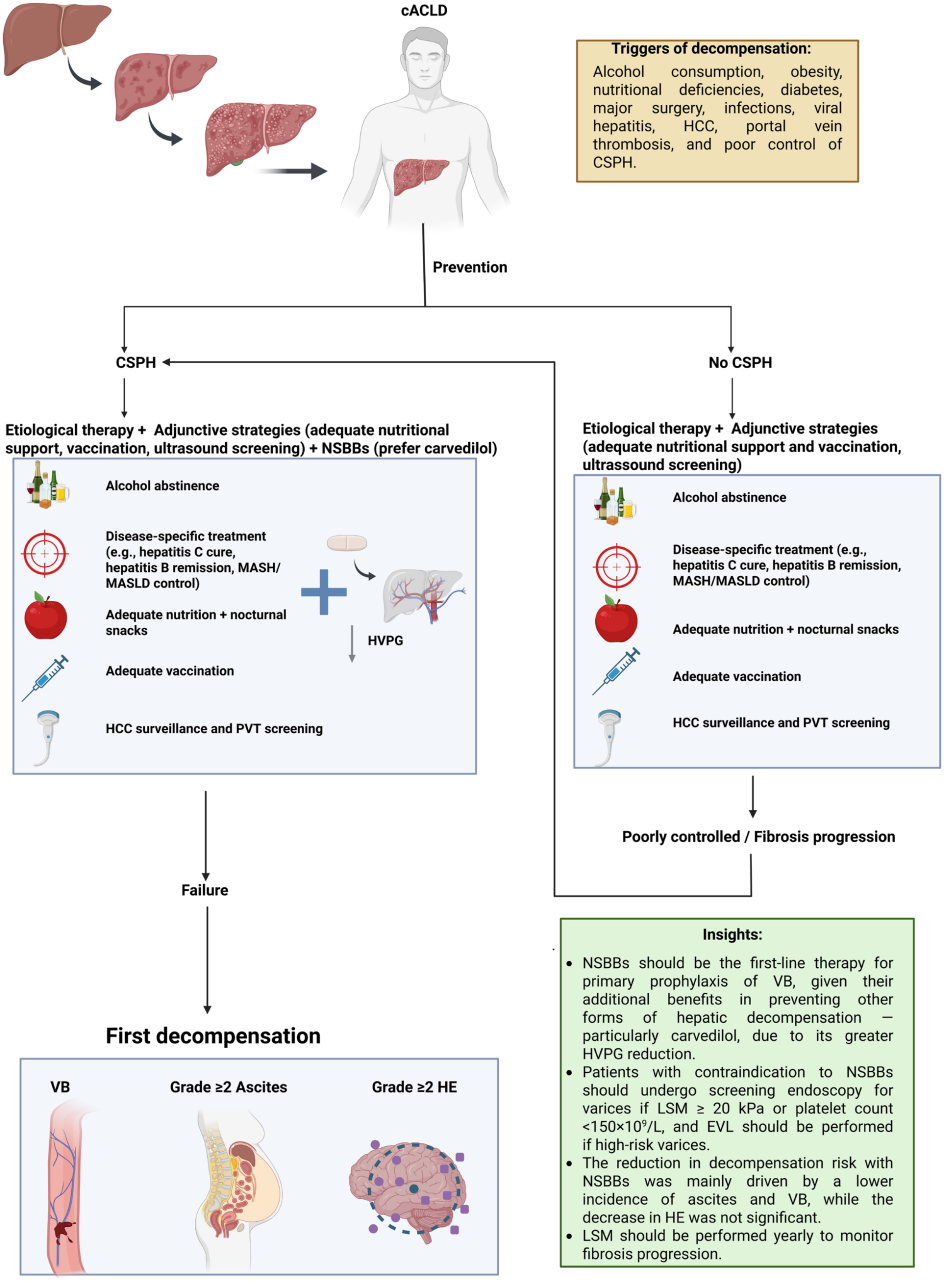
Schematic framework for preventing first decompensation. ACLD (Advanced Chronic Liver Disease), cACLD (Compensated Advanced Chronic Liver Disease), CSPH (Clinically Significant Portal Hypertension), dACLD (Decompensated Advanced Chronic Liver Disease), HE (Hepatic Encephalopathy), HCC (Hepatocellular Carcinoma), HRS‐AKI (Hepatorenal Syndrome–Acute Kidney Injury), HVPG (Hepatic Venous Pressure Gradient), LSM (Liver Stiffness Measurement), MASLD (Metabolic Dysfunction–Associated Steatotic Liver Disease), MASH (Metabolic Dysfunction–Associated Steatohepatitis), NSBBs (Non‐Selective Beta‐Blockers), PVT (Portal Vein Thrombosis), SBP (Spontaneous Bacterial Peritonitis), and VB (Variceal Bleeding).

The main adverse effect of EVL is bleeding from post‐ligation ulcers, occurring in about 5.5% of cases and potentially causing clinically significant or even fatal haemorrhage, especially in high‐MELD patients or when performed in emergency settings [41]. Some studies have suggested the use of proton pump inhibitors to mitigate this complication; however, a recent meta‐analysis demonstrated no significant benefit of this association [42] (Figure 2).

## 4 Liver Decompensation and Further Decompensation

When prevention of the first decompensation fails—often due to poor adherence, inadequate etiological control, or disease progression—the patient advances to dACLD. Notably, many individuals are diagnosed with ACLD only at their first decompensation episode, as cACLD is usually asymptomatic [4]. This gap in early detection is especially pronounced in low‐income settings, where access to diagnostic resources, such as elastography or invasive tests, is limited [43]. In such contexts, low‐cost non‐invasive tools (e.g., the FIB‐4 index) can help identify patients at risk by estimating advanced fibrosis [44]. Notably, in a large cohort from Vienna and Salzburg, a FIB‐4 threshold ≥ 1.75 identified individuals at increased risk of first decompensation (5‐year cumulative incidence: 7.6%), whereas risk was negligible among those with FIB‐4 < 1.75 (0.3%) [44]. These data suggest that inexpensive non‐invasive markers may also contribute to CSPH risk stratification. Nevertheless, when early identification fails and patients present at a later, decompensated stage, preventive strategies to mitigate further decompensation triggers should be initiated or continued, with renewed emphasis on strict sodium restriction, diet, and appropriate albumin replacement (6–8 g/L removed) after large‐volume paracentesis (> 5 L) [5].

**FIGURE 3:**
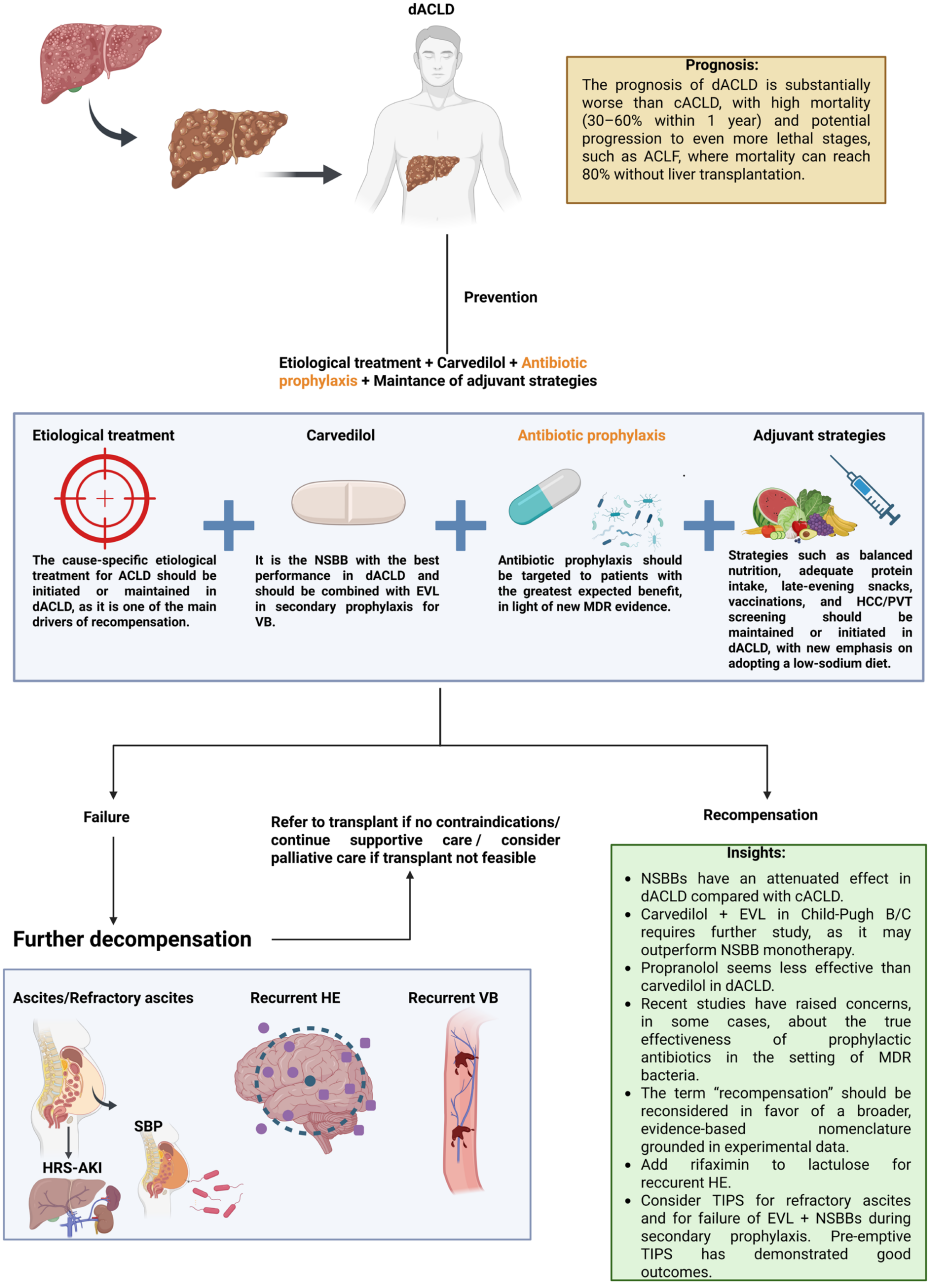
Schematic representation of strategies to prevent further decompensation. ACLD (Advanced Chronic Liver Disease), ACLF (Acute‐on‐Chronic Liver Failure), cACLD (Compensated Advanced Chronic Liver Disease), CSPH (Clinically Significant Portal Hypertension), dACLD (Decompensated Advanced Chronic Liver Disease), EVL (Endoscopic Variceal Ligation), HE (Hepatic Encephalopathy), HCC (Hepatocellular Carcinoma), HRS‐AKI (Hepatorenal Syndrome–Acute Kidney Injury), MDR (Multidrug‐Resistant), NSBBs (Non‐selective Beta‐Blockers), PVT (Portal Vein Thrombosis), SBP (Spontaneous Bacterial Peritonitis), TIPS (Transjugular Intrahepatic Portosystemic Shunt), and VB (Variceal Bleeding).

The main objective is to prevent further decompensation—considered a substage of dACLD, characterised by the recurrent or successive occurrence of decompensation events within a short period (refractory ascites, HRS‐AKI, SBP, recurrent HE and/or VB), which is associated with high mortality (30%–60% within 1 year) [4, 5, 6]. Many patients progress to ACLF, characterised by multiorgan failure and high short‐term mortality of up to 80% without liver transplantation [4]. For these reasons, preventing further decompensation is a critical step in managing dACLD, as it can substantially alter the disease course and improve survival (Figure 3).

### 4.1 Hepatic Recompensation

Hepatic recompensation should be pursued in dACLD, as it lowers mortality and HCC risk to a level similar to that of compensated cirrhosis [45]. Baveno VII defines it as partial reversal of cirrhotic structural and functional changes, closely linked to etiologic control [6]. Patients are considered recompensated when they achieve cure, viral suppression, or alcohol abstinence, with improvement in liver function (Child‐Pugh class A status)—evidenced by stabilisation of albumin, INR, and bilirubin—and sustained resolution of ascites (without diuretics), HE (without lactulose or rifaximin), and VB, all maintained for at least 12 months [6]. Even after recompensation, NSBBs generally should not be discontinued, as CSPH may persist and expose patients to a new decompensating event [6]. However, carefully selected recompensated patients with clear evidence that CSPH has regressed, stopping can be considered [5].

**FIGURE 4:**
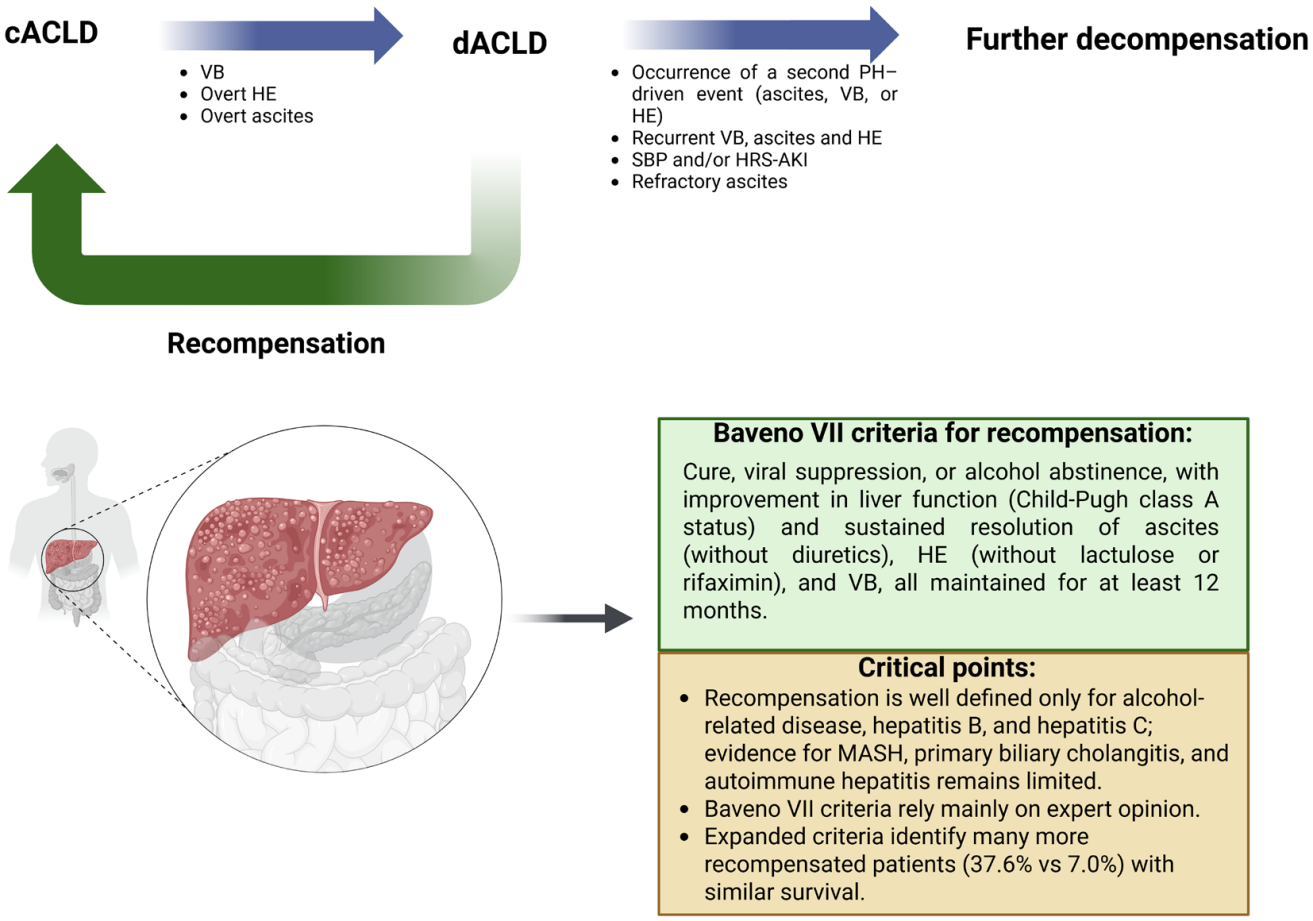
Transitions across the ACLD spectrum and current concepts of hepatic recompensation. CACLD (Compensated Advanced Chronic Liver Disease), dACLD (Decompensated Advanced Chronic Liver Disease), HE (Hepatic Encephalopathy), HRS‐AKI (Hepatorenal Syndrome–Acute Kidney Injury), MASH (Metabolic Dysfunction–Associated Steatohepatitis), PH (Portal Hypertension), SBP (Spontaneous Bacterial Peritonitis), and VB (Variceal Bleeding).

Hepatic recompensation is better understood in cases related to alcohol, hepatitis B virus, and hepatitis C virus [46]. In contrast, for etiologies such as MASH, primary biliary cholangitis, and autoimmune hepatitis, the understanding of recompensation remains limited, and the criteria are not well established [46]. This has raised concerns about the Baveno VII definitions, which rely on expert consensus rather than solid evidence, limiting their practical applicability. In addition, a study applying expanded and more flexible Baveno VII criteria allowing patients to be still on treatment with lactulose/rifaximin and/or a stable low dose of diuretics (anti‐aldosteronic drugs ≤ 200 mg/day and/or furosemide ≤ 25 mg/day with no increase in dose in the previous 12 months) identified a significantly higher number of recompensated patients (37.6% vs. 7.0%), with survival comparable to those meeting the original criteria [47]. This supports the notion that recompensation may occur earlier and that broader criteria better reflect the pathophysiological reality of these patients (Figure 4).

### 4.2 Considerations in the Use of NSBBs in dACLD

In dACLD, NSBBs show an apparently attenuated effect in preventing further compared to first decompensation [48]. Moreover, there appears to be a therapeutic window for NSBB use, as their benefit depends on the circulatory reserve and the cardiovascular system's ability to maintain adequate perfusion amid progressive splanchnic vasodilation [12, 49, 50]. From this perspective, in patients with dACLD, especially in the presence of refractory ascites, low mean arterial pressure, and impaired renal function, the use of NSBBs may be potentially harmful—a period during which the therapeutic window closes [12, 49, 50]. However, this tends to occur mainly when patients are already in the further decompensation substage, although one study suggested that propranolol may precipitate this state in patients with large‐volume ascites and Child‐Pugh B/C [51]. In this context, harm from NSBBs arises as reduced heart rate and contractility blunt compensatory responses to systemic vasodilation [12, 49, 50, 51].

When comparing propranolol to carvedilol in the prevention of further decompensation, current data suggest a more favourable profile for carvedilol. Recent cohort studies with adjusted analyses have shown better outcomes with carvedilol [37, 52], consistent with previous RCTs [53, 54], and limited to ascites in Sharma et al. [55]. Also, the lower efficacy of propranolol is supported by Wang et al. [56], which observed benefits only in patients with MELD < 9, and by Singh et al. [51], in which propranolol was associated with significantly lower 12‐month transplant‐free survival compared to EVL in decompensated patients (Child‐Pugh B/C) with large‐volume ascites (76% vs. 89.7%; *p* = 0.020). Similarly, meta‐regressions from a meta‐analysis comparing propranolol and EVL showed that the mortality benefit associated with propranolol occurred mainly in populations with a lower proportion of decompensated patients [40]. These findings may be explained by the greater HVPG reduction achieved with carvedilol even in dACLD without increasing adverse effects [57], whereas the more pronounced impact of propranolol on cardiac output may limit dose titration and contribute to loss of efficacy in dACLD, owing to progressive circulatory dysfunction (Table 1).

**TABLE 1:** Summary of studies reporting reduced propranolol efficacy in dACLD.

Author	Study design	Comparison	Number of patients	Main results
Fortea 2025 [33]	Retrospective cohort with adjusted multivariable analysis	Conventional NSBBs (propranolol + nadolol) vs. Carvedilol	284	In the conventional NSBBs group, more patients developed further decompensation (57.5% vs. 42.7%; *p* = 0.001), refractory ascites (20.1% vs. 9.3%; *p* = 0.002), and variceal bleeding (13.4% vs. 9.3%; *p* = 0.046). In addition, the combined endpoint of further decompensation or all‐cause mortality was significantly higher in the conventional NSBBs group (72.2% vs. 53.0%; *p* < 0.0001).
Singh 2022 [47]	RCT	Propranolol vs. EVL	160	In the propranolol group, more patients had worsening of ascites (15% vs. 5%; *p* = 0.030), developed refractory ascites (13.7% vs. 3.7%; *p* = 0.020), relapse of ascites (37.1% vs. 16.4%, *p* < 0.010), and acute kidney injury (26.2% vs. 12.5%; *p* = 0.020). In addition, transplant free survival was lower in the propranolol group (76% vs. 89.7%; *p* = 0.020).
Ismail 2025 [48]	Retrospective cohort with PSM.	Propranolol vs. Carvedilol	8640 (PSM)	In the PSM analysis, carvedilol consistently outperformed propranolol across both bleeding and non‐bleeding outcomes. Carvedilol was associated with a significantly lower risk of recurrent oesophageal VB (31.9% vs. 35.5%; RR 0.898, 95% CI 0.846–0.952; *p* < 0.001). It also reduced the risk of major further decompensation events, including new or worsening ascites (25.3% vs. 33.4%; RR 0.757, 95% CI 0.680–0.843; *p* < 0.001), SBP (5.4% vs. 7.9%; RR 0.680, 95% CI 0.574–0.807; *p* < 0.001), and HRS (5.9% vs. 8.0%; RR 0.734, 95% CI 0.623–0.865; *p* < 0.001). Carvedilol also demonstrated a lower incidence of HCC (4.3% vs. 6.2%; RR 0.701, 95% CI 0.578–0.852; *p* < 0.001) and markedly lower all‐cause mortality (20.2% vs. 31.6%; RR 0.640, 95% CI 0.593–0.690; *p* < 0.001). HE was the only outcome without a statistically significant difference (14.9% vs. 16.6%; RR 0.899, 95% CI 0.801–1.009; *p* = 0.071).
Kalambokis 2021 [49]	RCT	Propranolol vs. Carvedilol	96	Switching from propranolol to carvedilol (12.5 mg/day) resulted in a significant improvement in renal function, demonstrated by a progressive increase in GFR from 78.7 to 82.9 mL/min at 6 months (*p* = 0.001) and to 87.3 mL/min at 12 months (*p* < 0.001), along with an increase in renal blood flow (631 → 679 → 703 mL/min; *p* = 0.006 and *p* < 0.001, respectively) and a reduction in renal vascular resistance (*p* = 0.002 and *p* < 0.001). This hemodynamic improvement translated into a significantly lower risk of further decompensation at 2 years: 10.5% in the carvedilol group versus 35.9% in the propranolol group (*p* = 0.003), with carvedilol being an independent predictor of reduced risk (HR 0.832; *p* = 0.030).
Jachs 2023 [50]	RCT	Propranolol vs. Carvedilol (associated with EVL in both)	87	HVPG decreased more markedly in patients receiving carvedilol (median relative reduction: −20% [IQR: −29% to −10%] vs. –11% [−22% to −5%] with propranolol; *p* = 0.027). Cumulative rebleeding rates were significantly lower in the carvedilol group (*p* = 0.027), as was liver‐related mortality (*p* = 0.036). Moreover, the development or worsening of ascites occurred less frequently among patients treated with carvedilol (*p* = 0.012).
Sharma 2022 [51]	RCT	Propranolol vs. Carvedilol (associated with EVL in both)	48	Carvedilol showed a higher rate of hemodynamic HVPG response (72% vs. 47.8%; *p* = 0.047). The 1‐ and 3‐year rebleeding rates were similar between groups—16.0% and 24.0% with carvedilol versus 8.9% and 36.7% with propranolol (*p* = 0.457) — as were the 1‐ and 3‐year survival rates (94.7% and 89.0% for carvedilol; 100% and 79.8% for propranolol; *p* = 0.76). The only clinically relevant difference was new or worsening ascites, which was significantly more frequent in the propranolol group (69.5% vs. 40%; *p* = 0.040).
Wang 2024 [52]	Retrospective cohort with PSM and adjusted multivariable analyses	Propranolol vs. No use of NSBBs	332	Among patients with MELD ≤ 9, propranolol was linked to a reduced risk of further decompensation (sHR = 0.57; *p* = 0.021). Conversely, in patients with MELD > 9, propranolol was associated with an increased risk of further decompensation (sHR = 1.45; *p* = 0.044)
Süffert 2025 [36]	Meta‐analysis of 14 RCTs	Propranolol vs. EVL in the primary prophylaxis of oesophageal VB (compensated and decompensated cirrhosis)	1345	Although no difference in overall mortality was observed, meta‐regressions showed that a higher proportion of Child–Pugh A patients was associated with a more favourable effect of propranolol on all‐cause mortality (β = −1.48; 95% CI −2.75 to −0.21; *p* = 0.022). Consequently, cohorts with a greater proportion of Child–Pugh B/C patients tended to show the opposite pattern, with a possible association with higher mortality under propranolol. In addition, the presence of ascites correlated with better outcomes in the group treated with endoscopic variceal ligation (β = 1.23; 95% CI 0.17–2.29; *p* = 0.023).
Joshi 2025 [53]	Meta‐analysis of 7 RCTs	Propranolol vs. Carvedilol in their effect on HVPG	351	Carvedilol, when compared with propranolol, produced a greater reduction in HVPG (MD −0.76; *p* = 0.030), systemic vascular resistance (MD −190.6; *p* = 0.001), and mean arterial pressure (MD −3.65; *p* = 0.002).

Abbreviations: ACLD, Advanced Chronic Liver Disease; AKI, Acute Kidney Injury; dACLD, Decompensated Advanced Chronic Liver Disease; EVL, Endoscopic Variceal Ligation; GFR, Glomerular Filtration Rate; HCC, Hepatocellular Carcinoma; HE, Hepatic Encephalopathy; HRS, Hepatorenal Syndrome; HRS‐AKI, Hepatorenal Syndrome–Acute Kidney Injury; HVPG, Hepatic Venous Pressure Gradient; IQR, Interquartile Range; MELD, Model for End‐Stage Liver Disease; NSBBs, Non‐selective Beta‐Blockers; PSM, Propensity Score Matching; RCT, Randomised Controlled Trial; RR, Risk Ratio; SBP, Spontaneous Bacterial Peritonitis; VB Variceal Bleeding.

### 4.3 The Clinical Importance of EVL in dACLD

Although EVL is not traditionally recommended as a first‐line therapy for the primary prophylaxis of VB in patients with cACLD, its role becomes particularly relevant in the context of dACLD. Currently, major guidelines recommend EVL as the treatment of choice for secondary prophylaxis—that is, after a previous episode of oesophageal VB—in combination with NSBBs, particularly carvedilol [5, 6].

In the setting of primary prophylaxis in patients with dACLD, EVL is generally reserved for cases of intolerance to NSBBs [5, 6]. However, the CAVARLY trial [58] showed that combining carvedilol with EVL in patients with cirrhosis Child‐Pugh 7–13 and high‐risk varices was more effective than carvedilol alone in preventing first variceal bleeding and reducing mortality. The overall incidence of first variceal bleeding was significantly lower in the combination therapy group compared with NSBB monotherapy or EVL monotherapy (11.8% vs. 33.6% vs. 25.5%, respectively; *p* < 0.002). Likewise, 1‐year all‐cause mortality was significantly lower with combination therapy than with NSBB monotherapy or EVL monotherapy (6.3% vs. 20% vs. 14.5%, respectively; *p* = 0.012). This finding is biologically plausible, as the combination may enhance prevention of further decompensation by uniting the local mechanical effect of EVL with the reduction in HVPG achieved by NSBBs, particularly carvedilol. However, a meta‐analysis by Villanueva et al. [48] did not demonstrate a significant superiority of combination therapy over NSBB monotherapy for primary prophylaxis of VB in decompensated cirrhosis, although that analysis included only three RCTs using different NSBBs, which may have influenced the findings, as propranolol appears to have more limited efficacy in dACLD. Overall, despite the mechanistically appealing rationale for combining EVL with carvedilol, clear evidence of its advantage over NSBB monotherapy is still lacking.

### 4.4 Antibiotic Prophylaxis: Time to Rethink?

Antibiotic prophylaxis is also recommended by major guidelines in some cases [5, 6]. Primary SBP prophylaxis with norfloxacin (400 mg/day) or ciprofloxacin is indicated for patients with ascitic protein < 1.5 g/dL in combination with advanced liver dysfunction (Child‐Pugh ≥ B9 and serum bilirubin ≥ 3 mg/dL), or renal impairment (creatinine ≥ 1.2 mg/dL, blood urea nitrogen ≥ 25 mg/dL, or serum sodium < 130 mEq/L) [5]. In these patients, studies have demonstrated a significant reduction in SBP incidence and a delay in HRS with norfloxacin prophylaxis [5]. On the other hand, secondary prophylaxis of SBP should continue with oral norfloxacin until liver transplantation or clinical recompensation [5, 59]. For patients with acute gastrointestinal bleeding, in addition to hemodynamic stabilization and splanchnic vasoconstrictor use, short‐term antibiotic prophylaxis (typically with ceftriaxone for up to 5 days) is indicated, as this strategy reduces bacterial infections [5]. Finally, rifaximin has also shown promise in reducing further decompensation, especially post‐transjugular intrahepatic portosystemic shunt (TIPS) HE, but its role in primary SBP prophylaxis remains uncertain [60].

Importantly, routine use of antibiotic prophylaxis in dACLD, especially for those without high‐risk features, should not be recommended, as it may fuel the growing burden of multidrug‐resistant infections. Crucially, the evidence supporting antibiotic prophylaxis is increasingly questionable in light of new studies. Two major RCTs have further weakened support for primary prophylaxis of SBP [60, 61]. A recent trial showed that rifaximin offered no benefit for primary SBP prevention in severe cirrhosis with ascites, with no gains in survival or cirrhosis‐related outcomes [60]. In addition, the ASEPTIC trial, which is the largest to date evaluating primary antibiotic prophylaxis, showed that co‐trimoxazole did not improve overall survival in patients with cirrhosis and ascites [61]. Recent evidence also suggests that the clinical benefit of antibiotics is limited after upper gastrointestinal bleeding: a Bayesian meta‐analysis including 1322 cirrhotic patients found no reduction in mortality with prolonged antibiotic use compared with short courses or no prophylaxis [62], and a RCT in Child‐Pugh A patients also showed no benefit from ceftriaxone use following VB [63]. Furthermore, a cohort of more than 11 000 patients demonstrated an increased risk of SBP recurrence among those receiving secondary prophylaxis [64]. Therefore, these recommendations should be re‐evaluated in light of the current antimicrobial resistance landscape.

### 4.5 Key Recommendations for TIPS

Transjugular intrahepatic portosystemic shunt (TIPS) is a fundamental endovascular intervention for managing selected complications of dACLD, such as oesophageal and gastric VB and recurrent ascites. Preemptive TIPS is indicated in patients with dACLD who have acute VB at high risk of treatment failure (Child‐Pugh C 10–13 or Child‐Pugh B 8–9 with active bleeding), within 72 h of admission (ideally 24 h). It can also be used as salvage therapy in refractory haemorrhage. Overall, patients with more advanced hepatic dysfunction tend to derive greater benefit from pre‐emptive TIPS, given their substantially higher baseline risk of death and rebleeding. In an individual patient data meta‐analysis, pre‐emptive TIPS significantly improved survival among patients with active bleeding and Child–Pugh B8–9, whereas no survival benefit was observed in Child–Pugh B7 [65]. However, pre‐emptive TIPS reduced failure to control VB and subsequent rebleeding across Child–Pugh B strata (B7 and > 7 points) [65]. Emerging evidence also suggests a mortality benefit in particularly high‐risk phenotypes, including bleeding patients with ACLF and those with severe alcohol‐related hepatitis [66, 67].

TIPS additionally plays a crucial role in secondary prophylaxis when EVL plus NSBB therapy fails, and in the treatment of refractory ascites (particularly in those with MELD scores between 12 and 18 and Child‐Pugh B or early C) as well as hepatic hydrothorax. In patients with non‐malignant PVT, TIPS can restore portal flow and improve transplant eligibility [68, 69]. Despite its effectiveness, careful patient selection is essential due to the risks of HE and cardiac overload [68]. Finally, key predictors of poor outcomes after TIPS include advanced liver dysfunction (Child‐Pugh > 13, MELD > 18–20), recurrent HE, high bilirubin, active infection, cardiopulmonary disease, and age > 70 years [70].

## 5 Promising and Experimental Strategies

### 5.1 Statins

Statins deserve special attention in ACLD management, as their pleiotropic effects go beyond lipid lowering, targeting key mechanisms of fibrosis and PH [71]. Evidence shows they lower intrahepatic vascular resistance by enhancing endothelial NO bioavailability, promoting sinusoidal vasodilation [72, 73, 74]. Statins are considered safe in cACLD but should be used cautiously in dACLD due to the risk of adverse events, such as rhabdomyolysis [75]. Their anti‐inflammatory and antifibrotic properties [76, 77] may act synergistically with NSBBs, enhancing hepatic perfusion [77]. A recent meta‐analysis including both RCTs and observational studies showed that, although statins significantly reduced HVPG in the RCTs, this hemodynamic benefit did not translate into a clear reduction in decompensation events [78]. In contrast, the meta‐analysis reported an association with lower overall mortality in both RCTs and observational studies (moderate‐quality evidence) and a reduced incidence of HCC in observational cohorts (low‐quality evidence) [78]. Nevertheless, these effects may be modified by the specific statin prescribed, the duration and intensity of therapy, concomitant NSBBs use and population selection (e.g., inclusion of patients with earlier‐stage cirrhosis). For example, in a RCT by Kronborg et al. [79], atorvastatin did not improve clinical outcomes at 6 months of intervention. Currently, Baveno VII recommends statins for ACLD patients with a cardiovascular indication [6], while the definitive role of statins in cirrhosis still requires higher levels of evidence.

### 5.2 Albumin

The use of albumin is well established and standardised in several clinical settings in ACLD, including after large‐volume paracentesis (> 5 L; 6–8 g/L) to prevent post‐paracentesis circulatory dysfunction, in SBP (1.5 g/kg on day 1 and 1 g/kg on day 3) to reduce the risk of HRS and mortality, and in HRS‐AKI (1 g/kg, up to 100 g/day, for initial volume expansion followed by albumin in combination with vasoconstrictors) [5]. In addition, recent trials have shown potential benefits of chronic albumin infusions in dACLD, particularly through a reduction in liver decompensation in selected patients. The ANSWER trial showed that weekly albumin infusions (40 g) improved 18‐month survival, reduced complications (ascites recurrence, hyponatremia, SBP, HRS), and decreased the need for large‐volume paracentesis compared with standard medical therapy [80]. By contrast, the MACHT study evaluated long‐term administration of midodrine plus albumin (40 g every 15 days) in decompensated patients listed for liver transplantation and found no reduction in liver‐related complications or mortality at 1 year [81]. Therefore, the role of chronic albumin infusion in dACLD remains controversial.

### 5.3 Anticoagulation

The CIRROXABAN trial evaluated a reduced dose of rivaroxaban (10 mg once daily) versus placebo in 90 patients with cirrhosis Child‐Pugh 7–10 and PH over a 24‐month period [82]. The study suggested that rivaroxaban may reduce the risk of portal hypertension–related complications or death/liver transplantation, with a trend toward improved event‐free survival (*p* = 0.058) and no significant difference in major bleeding events between groups. In a post hoc analysis, a significant benefit was observed in patients with Child–Pugh B7 cirrhosis (HR: 0.258, 95% CI 0.074–0.90), suggesting rivaroxaban may improve PH‐complication free survival [82].

### 5.4 Therapies for MASH‐Related ACLD

FGF21 analogs are considered a promising therapeutic option for MASH‐cirrhosis as it can potentially reverse fibrosis and improve inflammation [83]. Drugs such as efruxifermin, pegbelfermin, and pegozafermin have demonstrated a significant increase in the proportion of MASH patients achieving improvement in fibrosis. In the SIMMETRY trial, which included patients with MASH and biopsy‐confirmed compensated cirrhosis Child‐Pugh 5 or 6, 50‐mg weekly efruxifermin for 96 weeks led to reversal of cirrhosis in 29% vs. 11% in the placebo group [83].

Resmetirom, a selective thyroid hormone receptor‐β agonist, was the first MASH therapy to be approved by the US Food & Drug Administration as it stimulates lipid metabolism, improves inflammation and reduces fibrosis [84]. Recent evidence shows that, in 122 patients with MASH‐related cACLD, 2 years of treatment reduced noninvasive biomarkers, decreased liver stiffness, and improved parameters related to PH [85]. At baseline, 63% of patients met probable/definitive CSPH criteria (Baveno VII); however, at 1 and 2 years, 20% and 28%, respectively, no longer fulfilled CSPH criteria [85].

In patients with cACLD due to MASH, semaglutide (a GLP‐1 receptor agonist) did not achieve higher rates of MASH resolution or fibrosis improvement compared with placebo [86]. Moreover, available data have not shown a consistent reduction in cirrhosis‐related complications [86, 87]. Overall, GLP‐1 receptor agonists appear to be generally well tolerated in cACLD; however, their use in decompensated cirrhosis is not supported by current evidence.

## 6 Conclusions

The prevention of first and further decompensation in patients with ACLD requires a continuous multidisciplinary approach that should be systematically integrated into the clinical management. In the context of preventing new decompensations, carvedilol has emerged as the most effective therapeutic strategy, including in cases of dACLD, since propranolol shows even more limited efficacy at this stage—an aspect not fully recognised in the past. Future investigations should focus on deepening the understanding of the combined use of EVL and carvedilol in dACLD, considering the attenuated effect of NSBBs in this phase, as well as reassessing the need for antibiotic prophylaxis in light of the current scenario of increasing antimicrobial resistance. Moreover, it is essential to explore strategies of clinical recompensation and the potential role of statins in preventing hepatic decompensation. Ultimately, advancing a more precise and stage‐adapted model of care will be key to improving long‐term outcomes in ACLD.

## Funding

The Article Processing Charge for the publication of this research was funded by the Coordenação de Aperfeiçoamento de Pessoal de Nível Superior ‐ Brasil (CAPES) (ROR identifier: 00x0ma614).

## Conflicts of Interest

The authors declare no conflicts of interest.

## Data Availability

Data sharing not applicable to this article as no datasets were generated or analysed during the current study.
